# YM750, an ACAT Inhibitor, Acts on Adrenocortical Cells to Inhibit Aldosterone Secretion Due to Depolarization

**DOI:** 10.3390/ijms232112803

**Published:** 2022-10-24

**Authors:** Hiroki Shimada, Shuko Hata, Yuto Yamazaki, Yuri Otsubo, Ikuko Sato, Kazue Ise, Atsushi Yokoyama, Takashi Suzuki, Hironobu Sasano, Akira Sugawara, Yasuhiro Nakamura

**Affiliations:** 1 Division of Pathology, Faculty of Medicine, Tohoku Medical and Pharmaceutical University, 1-15-1 Fukumuro, Miyagino-ku, Sendai 983-8536, Miyagi, Japan; 2 Department of Pathology, Tohoku University Graduate School of Medicine, 2-1 Seiryo-machi, Aoba-ku, Sendai 980-8575, Miyagi, Japan; 3 Department of Molecular Endocrinology, Tohoku University Graduate School of Medicine, 2-1 Seiryo-machi, Aoba-ku, Sendai 980-8575, Miyagi, Japan

**Keywords:** ACAT1, YM750, aldosterone, CYP11B2, NURR1, immunohistochemistry, ZG

## Abstract

Primary aldosteronism (PA) is considered the most common form of secondary hypertension, which is associated with excessive aldosterone secretion in the adrenal cortex. The cause of excessive aldosterone secretion is the induction of aldosterone synthase gene (*CYP11B2*) expression by depolarization of adrenocortical cells. In this study, we found that YM750, an Acyl-coenzyme A: cholesterol acyltransferase (ACAT) inhibitor, acts on adrenocortical cells to suppress *CYP11B2* gene expression and aldosterone secretion. YM750 inhibited the induction of *CYP11B2* gene expression by KCl stimulation, but not by angiotensin II and forskolin stimulation. Interestingly, YM750 did not inhibit KCl-stimulated depolarization via an increase in intracellular calcium ion concentration. Moreover, ACAT1 expression was relatively abundant in the zona glomerulosa (ZG) including these CYP11B2-positive cells. Thus, YM750 suppresses *CYP11B2* gene expression by suppressing intracellular signaling activated by depolarization. In addition, ACAT1 was suggested to play an important role in steroidogenesis in the ZG. YM750 suppresses *CYP11B2* gene expression and aldosterone secretion in the adrenal cortex, suggesting that it may be a potential therapeutic agent for PA.

## 1. Introduction

Aldosterone is synthesized from cholesterol by the action of steroidogenic enzymes in the adrenal cortex. Aldosterone synthase (*CYP11B2*) is the rate-limiting enzyme in aldosterone synthesis. Primary aldosteronism (PA), a condition characterized by excess production of aldosterone by the adrenal cortex, is a common cause of secondary hypertension [1,2,3]. The causes of aldosterone overproduction in the adrenal cortex include idiopathic hyperaldosteronism (IHA), aldosterone-producing adenomas (APAs), and adrenocortical carcinoma, among others. In 2022, the World Health Organization classified aldosterone-producing lesions in the adrenal cortex based on the area where *CYP11B2* was expressed [4]. In particular, aldosterone-producing nodules (APNs), aldosterone-producing micronodules (APMs), multiple APNs, multiple APMs, and aldosterone-producing diffuse hyperplasia (APDH) were reported to be associated with the development of IHA [5]. Mutations in the *CACNA1D*, *ATP1A1*, and *ATP2B3* genes have been identified in the adrenal *CYP11B2*-positive regions, such as APNs/APMs (formerly known as aldosterone-producing cell clusters) and APDH (formerly known as diffuse hyperplasia) [6,7,8]. *CYP11B2* expression and aldosterone secretion caused by cellular depolarization are linked to PA pathogenesis [9,10,11,12,13]. Therefore, it is essential to develop therapeutic agents that target depolarization in APNs, APMs, and APDH for the treatment of PA.

Drugs that act on the adrenal cortex and suppress aldosterone secretion are currently still under development; however, macrolides that suppress aldosterone secretion in APAs associated with *KCNJ5* mutations have been reported [14]. Additionally, *CYP11B2* inhibitors have also been developed [15]. The recently elucidated relationship between aldosterone secretion in APAs and the adrenal cortex and the expression of cholesterol-metabolizing enzymes including acyl-coenzyme A: cholesterol acyltransferase 1(ACAT1) [16] is useful in analyzing cholesterol and aldosterone secretion.

ACAT is a cholesterol-metabolizing enzyme, expressed as ACAT1 in macrophages and adrenal glands and as ACAT2 in the liver [17]. ACAT converts cholesterol to cholesteryl esters. In the adrenocortical cells, ACAT1 temporarily converts cholesterol to cholesteryl esters, which are stored as a source of cholesterol for the synthesis of steroid hormones [18].

However, the relationship between ACAT activity and adrenocortical steroid synthesis remains unclear. Additionally, the expression status of *ACAT1* in APNs, APMs, and APDH has not been studied in detail.

In this study, we demonstrated that YM750, an ACAT1 inhibitor, suppressed aldosterone synthesis, suggesting its potential as a novel therapeutic agent for PA. In addition, we reported the detailed expression status of *ACAT1* in *CYP11B2*-positive aldosterone-producing cells.

## 2. Results

### 2.1. Effect of YM750 on CYP11B2 Expression Induced by Various Stimuli

**Figure 1:**
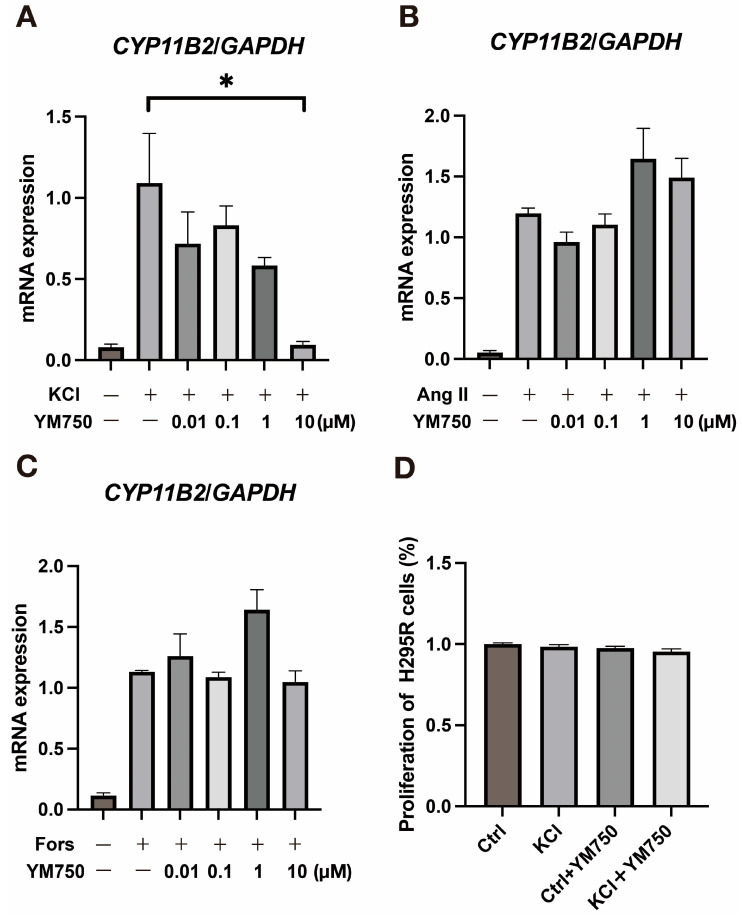
Effect of YM750 on *CYP11B2* expression induced by various stimuli. (**A**) Effect of YM750 on *CYP11B2* expression induced by KCl (20 mM) stimulation. (**B**) Effect of YM750 on *CYP11B2* expression induced by angiotensin II (100 nM) stimulation. (**C**) Effect of YM750 on *CYP11B2* expression induced by forskolin (10 µM) stimulation. (**D**) Effect of YM750 (10 µM) on H295R cell proliferation. * *p* < 0.05, n = 3.

We first examined the effect of YM750 on *CYP11B2* expression. The results showed that YM750 suppressed KCl-stimulated *CYP11B2* expression but showed no effect on angiotensin II (Ang II)- and forskolin-stimulated *CYP11B2* expression (Figure 1A–C). Cell viability was unaffected by YM750, indicating that suppression of *CYP11B2* expression by YM750 was not mediated by cytotoxicity (Figure 1D).

### 2.2. Effect of YM750 on the Expression of Various Steroidogenic Enzymes and CYP11B2 Related Genes

**Figure 2:**
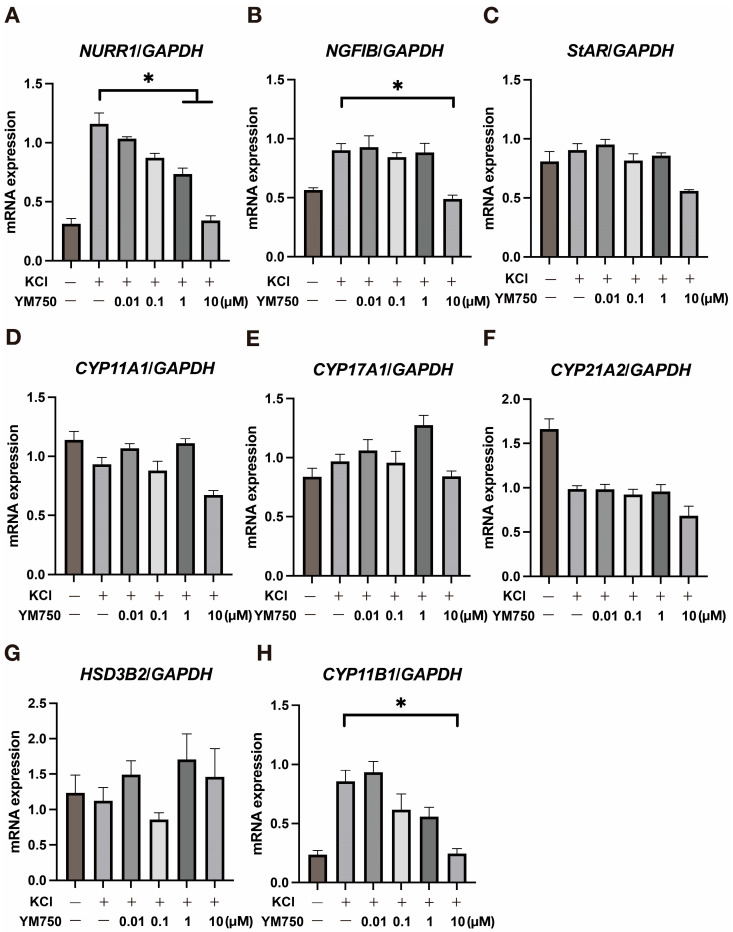
Effect of YM750 on the expression of various steroidogenic enzymes and *CYP11B2* related genes. Effect of YM750 on (**A**) *NURR1*, (**B**) *NGFIB*, (**C**) *StAR*, (**D**) *CYP11A1*, (**E**) *CYP17A1*, (**F**) *CYP21A2*, (**G**) *HSD3B2* and (**H**) *CYP11B1* expression induced by KCl stimulation. * *p* < 0.05, n = 3.

YM750 suppressed KCl-stimulated induction of *CYP11B2* expression. The effect of YM750 on *NURR1* and *NGFIB* genes in the adrenal glands was also examined. NURR1 and NGFIB are orphan nuclear receptors that induce transcription factors essential for *CYP11B2* expression [19]. The expression of *NURR1*, *NGFIB*, and *CYP11B1* was suppressed in the group treated with high concentration of YM750 (Figure 2A,B). In contrast, YM750 showed no significant effect on the expression of genes encoding steroidogenic enzymes (Figure 2C–H).

### 2.3. Effect of YM750 on Aldosterone Secretion

**Figure 3:**
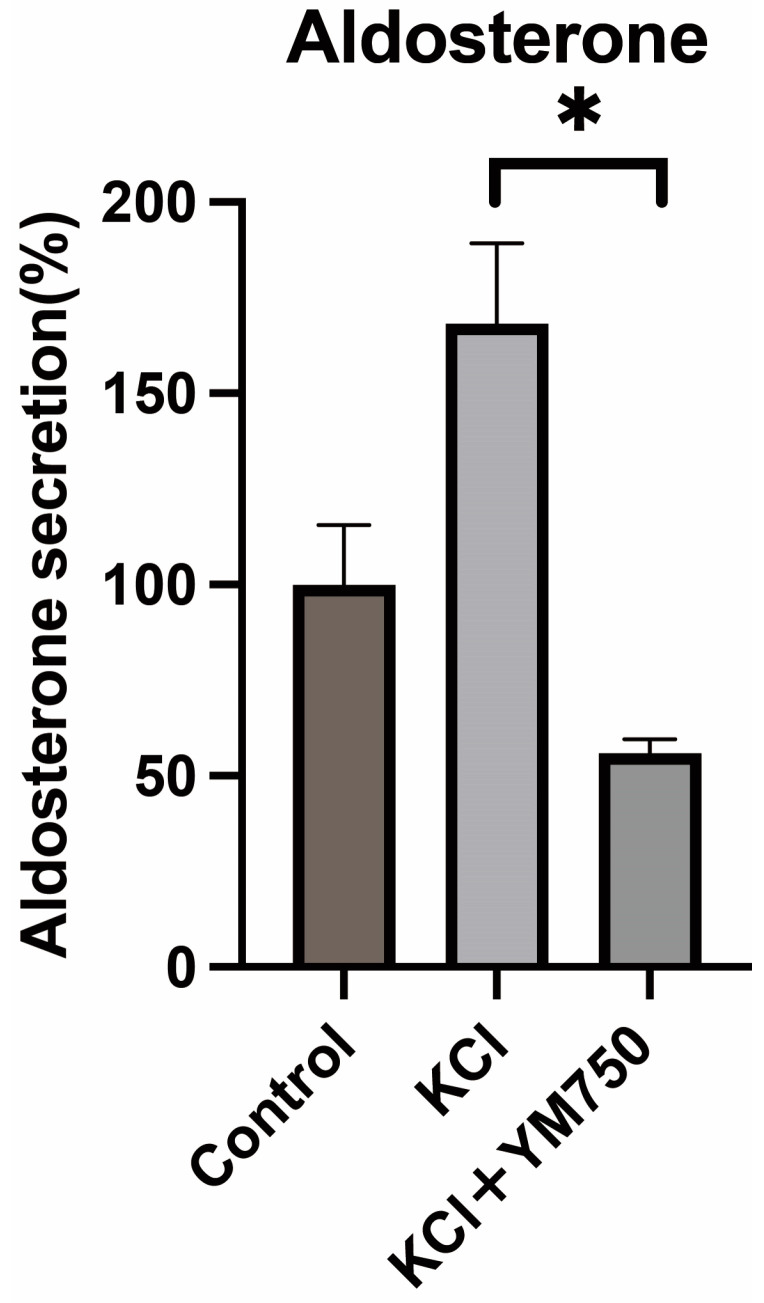
Effect of YM750 (10 mM) on aldosterone secretion by KCl (20 mM) stimulation. * *p* < 0.05, n = 4.

H295R cells were incubated with YM750 and KCl for 24 h, and aldosterone concentration in the supernatant was measured using the Aldosterone ELISA kit. The results showed that aldosterone secretion was significantly suppressed in the YM750-treated group (Figure 3). These results suggested that YM750 suppresses aldosterone secretion.

### 2.4. Effect of YM750 on Intracellular Calcium Concentration

**Figure 4:**
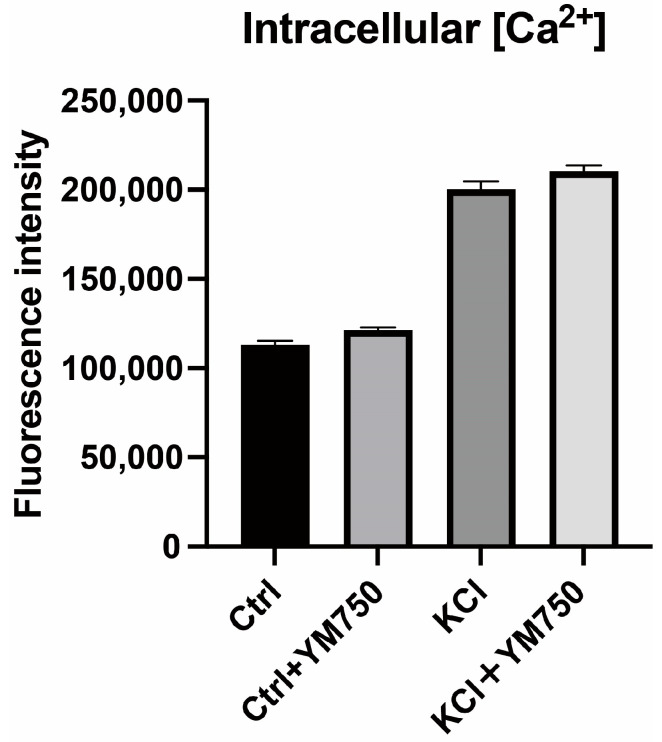
Effect of YM750 (10 mM) on intracellular calcium concentration by KCl (20 mM) stimulation. n = 4.

As described earlier, YM750 suppressed *CYP11B2* expression and aldosterone secretion in KCl-stimulated H295R cells. The effect of YM750 on intracellular calcium concentration was examined. The results showed that YM750 did not alter the intracellular calcium concentration (Figure 4). This suggests that the suppression of *CYP11B2* expression by YM750 is not mediated by a decrease in intracellular calcium.

### 2.5. Expression and Localization of ACAT1 in the Adrenal Cortex

**Figure 5:**
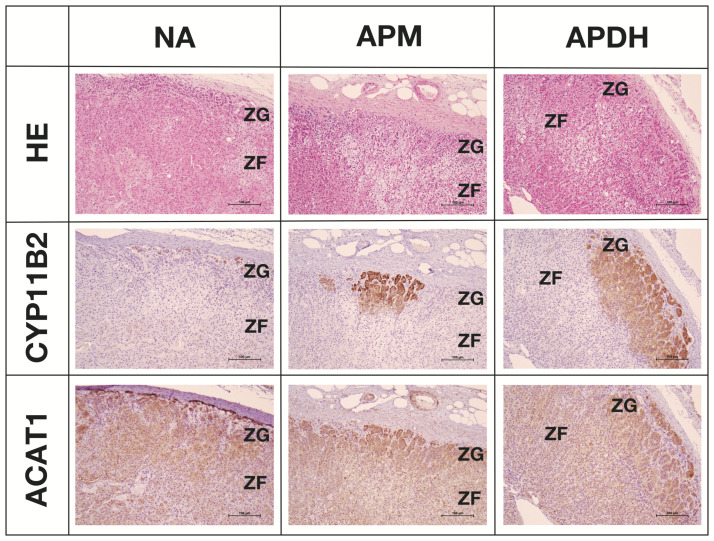
Expression and localization of *ACAT1* in the adrenal cortex. HE: hematoxylin and eosin stains, CYP11B2: IHC using anti-CYP11B2 antibody, ACAT1: IHC using anti-ACAT1 antibody, NA: normal adrenal, APM: aldosterone-producing micronodule, APDH: aldosterone-producing diffuse hyperplasia. Scale in the image indicates 100μm.

We examined *ACAT1* expression in the adrenal cortex. We assessed the localization of *CYP11B2* and *ACAT1* expression in APNs, APMs, and APDH (Figure 5). The results showed that *ACAT1* is expressed in all layers of the adrenal cortex. *ACAT1* expression was particularly abundant in the zona glomerulosa (ZG), including *CYP11B2*-positive cells.

## 3. Discussion

To the best of our knowledge, this is the first study to demonstrate that YM750 suppresses *CYP11B2* expression and aldosterone secretion by inhibiting downstream calcium signaling and inducing expression of *NURR1*.

YM750 reduces the concentration of intracellular cholesteryl esters by inhibiting ACAT1 [20]. The results from this study showed that YM750 suppressed only KCl-stimulated *CYP11B2* gene expression but showed no effect on Ang II- and forskolin-stimulated *CYP11B2* expression. Additionally, YM750 did not suppress calcium ion influx into the cells. KCl stimulation depolarizes H295R cells and induces *CYP11B2* expression via activation of intracellular calcium signaling [7]. Ang II binds to the angiotensin type 1 receptor and activates the phosphoinositide 3-kinase (PI3K) signaling pathway, MEK-ERK signaling cascade, and intracellular calcium signaling pathway. Forskolin activates adenylate cyclase, which in turn activates the cAMP-PKA signaling pathway. Ang II and forskolin induce *CYP11B2* expression via activation of transcription factors such as NURR1 and cyclic AMP response element-binding protein [7]. These results suggest that YM750 suppressed intracellular calcium signaling activated by KCl-stimulated depolarization but showed no effect on the PI3K and cAMP-PKA signaling pathways, thus resulting in suppression of *CYP11B2* expression specifically.

It has also been reported that *CYP11B2* expression is regulated by transcription factors such as NURR1 and NGFIB [21]. NURR1 and NGFIB bind to the promoter region of *CYP11B2* and regulate gene expression [19]. NGFIB binds to the promoter region of *HSD3B2* and regulates gene expression [22]. YM750 suppressed *NURR1* and *NGFIB* expression at high concentrations. This suggests that the suppression of NURR1 and NGFIB. This suggests that ACAT1 is associated with suppressed *CYP11B2* expression via repression of NURR1 and NGFIB. NURR1 primarily regulates *CYP11B2* expression, while NGFIB regulates the expression of other steroidogenic genes [21,23,24]. Therefore, it is hypothesized that YM750 selectively suppresses *NURR1* expression as compared to *NGFIB* expression, thereby suppressing *CYP11B2* expression specifically. In addition, TM750 suppressed *CYP11B1* expression. It is known that H295R cells share common signal pathway related to the expression of *CYP11B1* and *CYP11B2* [25]. Therefore, it is postulated that *CYP11B1* similarly responded to KCl-stimulated depolarization, compatible with inhibition of *CYP11B1* expression by YM750. However, it is speculated that YM750 does not necessarily inhibit CYP11B1 in vivo because CYP11B1 expressed in the ZF but not in the ZG would be mainly regulated by ACTH-stimulated cAMP-PKA signaling pathway rather than potassium-stimulated depolarization. In addition, it remains unclear how YM750 effect on metabolism and cholesterol levels when it is clinically used as an aldosterone secretion inhibitor. Therefore, further studies are required for clarification in the future.

**Figure 6:**
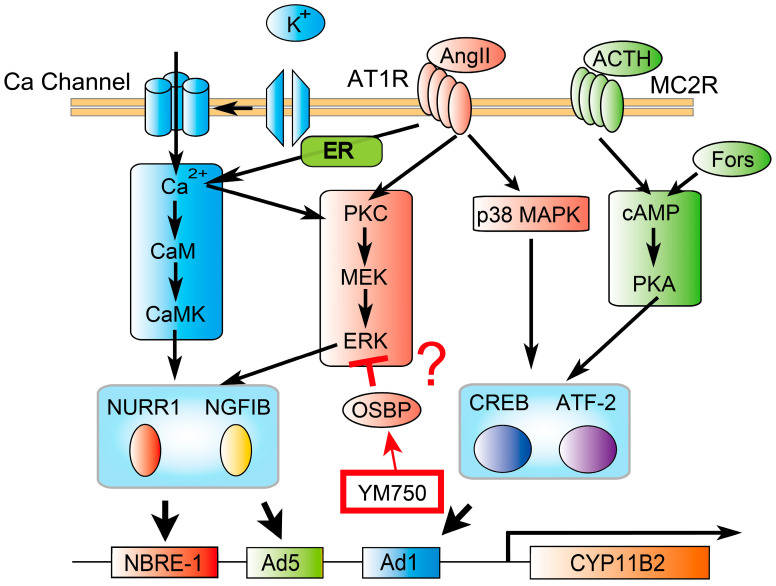
Graphical summary based on our current results.

The results from this study also suggest that ACAT1 inhibition suppressed intracellular cholesterol accumulation. Cholesterol accumulation activates oxysterol-binding protein (OSBP) and suppresses the activation of extracellular signal-regulated protein kinase (ERK1/2) and mitogen-activated protein kinase (MAPK) signaling pathways [26]. OSBP is expressed in the adrenal gland (Human Protein Atlas: https://www.proteinatlas.org/ENSG00000110048-OSBP#gene_information, accessed on 5 October 2022 [27]) and may be affected by ACAT inhibitors. ERK1/2 activation induces *CYP11B2* expression via *NURR1* and *NGFIB* expression [22]. Although it can be hypothesized that ACAT1 inhibition suppresses *CYP11B2* expression via activation of OSBP, confirmatory evidence in support of this hypothesis is yet to be established (Figure 6). Therefore, it awaits future investigation for clarification on this point. ATR-101, an ACAT1 inhibitor, is effective in the treatment of Cushing’s syndrome in dogs [28]. ATR-101 also moderately decreased aldosterone secretion in dogs, suggesting that ACAT1 inhibition may affect steroidogenesis in the adrenal gland. 

In this study, we demonstrated that *ACAT1* is widely expressed in the adrenal cortex and is abundant in the ZG, including APNs. *ACAT1* is also expressed in APAs and cortisol-producing adenomas [16,29], suggesting that ACAT1 participates in steroid production in tumor tissues. *NURR1* is also widely expressed in the ZG [30], indicating an association between ACAT1 and NURR1 in steroidogenesis. Additionally, APMs and APDH with a higher concentration of *CYP11B2*-positive cells were involved in aldosterone secretion to a greater extent as compared to APNs [8]. Therefore, these results suggest that *ACAT1* expression plays an important role in excess aldosterone production in APAs, APMs, and APDH.

## 4. Materials and Methods

### 4.1. Drugs

Angiotensin II (Sigma, Burlington, MA, USA) was dissolved in phosphate-buffered saline (PBS) containing 0.1% bovine serum albumin (BSA; Sigma, St. Louis, MO, USA). Potassium chloride (KCl; Wako, Osaka, Japan) was dissolved in water. Forskolin was dissolved in ethanol (Wako Pure Chemical Industries, Osaka, Japan). YM750 (Cayman Chemical, Ann Arbor, MI, USA) was dissolved in dimethyl sulfoxide (Wako).

### 4.2. Cell Culture

H295R cells derived from human adrenocortical carcinoma were purchased from American Type Culture Collection and cultured in Dulbecco’s Modified Eagle Medium (DMEM)/Ham’s F-12 nutrient medium (Wako) supplemented with 10% fetal bovine serum (FBS, Life Technologies, Edina, MN, USA), 1.25 mg/mL BSA (Sigma), 1× insulin-transferrin-selenium solution (Life Technologies), 5.35 mg/mL linoleic acid (Sigma), and 1× penicillin-streptomycin solution (Wako). For angiotensin II-, KCl-, forskolin-, and YM750-induced stimulation, cells were cultured in DMEM (Wako) containing 1% charcoal-treated FBS with penicillin-streptomycin. The culture and stimulation methods were based on previous studies [31,32,33].

### 4.3. RNA Extraction, cDNA Synthesis, and Quantitative Reverse Trascription Polymerase Chain Reaction (RT-qPCR)

Total RNA was extracted using Sepasol-RNA I Super G (Nacalai Tesque, Kyoto, Japan) and cDNA was synthesized using the Prime Script RT Master Mix (TAKARA, Tokyo, Japan). Real-time RT-qPCR was performed using the Thermal Cycler Dice Real-Time System (TP800, Takara, Kusatsu, Japan) along with the THUNDERBIRD^®^ Probe qPCR Mix (Toyobo, Osaka, Japan) for analyzing *CYP11B2* and *HSD3B2* and KAPA SYBR FAST qPCR kit (KAPA BIOSYSTEMS, Wilmington, MA, USA) for analyzing other genes. Primer sequences and TaqMan probes are listed in Table 1.

### 4.4. Cell Viability Assay

Two days after plating 2.5 × 10^4^ H295R cells into 96-well plates, the cells were incubated with and without 10 mM YM750 for 24 h and 20 mM KCl for 6 h. Cell viability was measured using the Cell Counting Kit-8 (Dojindo, Kumamoto, Japan) following the manufacturer’s instructions.

### 4.5. Aldosterone EIA

Two days after plating 3.0 × 10^5^ H295R cells into 24-well plates, the cells were incubated with and without 10 mM YM750 and 20 mM KCl for 24 h. Aldosterone and cortisol concentrations in the media were measured using the Aldosterone ELISA Kit (Cayman Chemical, Ann Arbor, MI, USA) following the manufacturer’s instructions.

### 4.6. Intracellular Calcium Ion Concentration Assay

Two days after plating 2.5 × 10^4^ H295R cells into 96-well plates, the cells were incubated with and without 10 mM YM750 for 24 h. The cells were then loaded with Fluo 4-AM (Dojindo, Rockville, MD, USA; 5 mg/mL) in the presence of 1.25 mM probenecid (Dojindo) and 0.04% Pluronic F-127 (Dojindo) for 1 h. Cells were then washed with PBS and the recording medium containing 1.25 mmol/L probenecid and 20 mM KCl was added to the media. Changes in intracellular calcium concentration were determined by measuring the fluorescence intensity (excitation wavelength, 485 nm; emission wavelength, 535 nm).

### 4.7. Human Adrenal Tissue Samples for Immunohistochemical Analysis

Three non-pathological adrenal glands (normal adrenal (NA) tissues), three multiple APMs (APM tissues), and three APDH (APDH tissues) were selected for immunohistochemical analysis based on information retrieved from the surgical pathology files from Tohoku University Hospital (Sendai, Japan). NA tissues of nephrectomy cases due to renal carcinoma were harvested and subsequently evaluated to confirm the absence of neoplastic invasion, necrosis, and other histopathological abnormalities. The research protocols were approved by the Ethics Committee of Tohoku University Graduate School of Medicine (Sendai, Japan). All patients read and signed informed consent forms that clearly stated the methodologies for the use of tissue samples and clinical data for diagnostic and scientific purposes in the present study. The research protocol was approved by the Institutional Review Board of the Tohoku University School of Medicine (approval number 2020-1-705).

### 4.8. Immunohistochemical Analysis

Immunohistochemical analysis using hematoxylin and eosin stains was performed on 3 μm thick tissue sections prepared from formalin-fixed, paraffin embedded blocks. The protocols for immunohistochemistry used in this study are summarized in Table 2 [16,34].

### 4.9. Statistical Analysis

All data are presented as mean ± standard error of the mean. Statistical analyses were performed using Welch’s analysis of variance using GraphPad Prism 9.

## 5. Conclusions

In this study, we demonstrated that YM750, an ACAT1 inhibitor, suppressed *CYP11B2* expression and aldosterone secretion in H295R cells. Additionally, *ACAT1* expression was confirmed in the ZG of normal adrenal glands and in non-neoplastic regions of PA. The results from this study suggest that YM750 may be a potential therapeutic agent for PA.

## Figures and Tables

**Table 1:** Primer set using RT-PCR.

Gene	Forward Primer (5′→3′)	Reverse Primer (3′→5′)
*CYP11B2*	GGCAGAGGCAGAGATGCTG	CTTGAGTTAGTGTCTCCACCAGGA
*NURR1*	AGAGAAGATCCCTGGCTTCG	CAAGACCACCCCATTGCAAAA
*NGFIB*	CCTGGAGCTCTTCATCCTCC	TGTCAATCCAGTCCCCGAAG
*StAR*	GCATCCTTAGCAACCAAGAG	TCACTTTGTCCCCATTGTCC
*CYP11A1*	TTCCGCTTTGCCTTTGAGTC	TGGCATCAATGAATCGCTGG
*CYP17A1*	CAGAATGTGGGTTTCAGCCG	CTCACCGATGCTGGAGTCAA
*CYP21A2*	AGACTACTCCCTGCTCTGGA	CTCATGCGCTCACAGAACTC
*HSD3B2*	GCGGCTAATGGGTGGAATCTA	CATTGTTGTTCAGGGCCTCAT
*CYP11B1*	GGCAGAGGCAGAGATGCTG	TCTTGGGTTAGTGTCTCCACCTG
*GAPDH*	ATCCCATCACCATCTTCCAG	ATGAGTCCTTCCACGATACC
**Gene**	**Probe**	
*CYP11B2*	[6-FAM]CTGCACCACGTGCTGAAGCACT[TAMPA6-FAM]	
*HSD3B2*	[6-FAM]TGATACCTTGTACACTTGTGC[TAMPA6-FAM]	
*CYP11B1*	[6-FAM]TGCTGCACCATGTGCTGAAACACCT[TAMRA-6-FAM]	

**Table 2:** Method of immunohistochemical analysis.

Primary Antibody	Dilution	Species	Clone	Company	Antigen Retrieval Treatment
ACAT1	1:100	Rabbit	Monoclonal (EPR10359)	Abcam	AC 121 °C 5 min, pH = 9 Buffer
CYP11B2	1:500	Mouse	Monoclonal	Gomez-Sanchez et al., 2014 [34]	AC 121 °C 5 min, pH high Buffer

## Data Availability

The data presented in this study are openly available online.
